# Neurons in primate prefrontal cortex signal valuable social information during natural viewing

**DOI:** 10.1098/rstb.2019.0666

**Published:** 2021-01-11

**Authors:** Geoffrey K. Adams, Wei Song Ong, John M. Pearson, Karli K. Watson, Michael L. Platt

**Affiliations:** 1 Department of Neuroscience, Perelman School of Medicine, University of Pennsylvania, Philadelphia, PA, USA; 2 Department of Psychology, School of Arts and Sciences, University of Pennsylvania, Philadelphia, PA, USA; 3 Marketing Department, Wharton School of Business, University of Pennsylvania, Philadelphia, PA, USA; 4 Department of Biostatistics and Bioinformatics, Duke University, Durham, NC, USA; 5 Institute of Cognitive Science, University of Colorado at Boulder, Boulder, CO, USA

**Keywords:** neuroethology, PFC, social gaze

## Abstract

Information about social partners is innately valuable to primates. Decisions about which sources of information to consume are highly naturalistic but also complex and place unusually strong demands on the brain's decision network. In particular, both the orbitofrontal cortex (OFC) and lateral prefrontal cortex (LPFC) play key roles in decision making and social behaviour, suggesting a likely role in social information-seeking as well. To test this idea, we developed a ‘channel surfing' task in which monkeys were shown a series of 5 s video clips of conspecifics engaged in natural behaviours at a field site. Videos were annotated frame-by-frame using an ethogram of species-typical behaviours, an important source of social information. Between each clip, monkeys were presented with a choice between targets that determined which clip would be seen next. Monkeys' gaze during playback indicated differential engagement depending on what behaviours were presented. Neurons in both OFC and LPFC responded to choice targets and to video, and discriminated a subset of the behaviours in the ethogram during video viewing. These findings suggest that both OFC and LPFC are engaged in processing social information that is used to guide dynamic information-seeking decisions.

This article is part of the theme issue ‘Existence and prevalence of economic behaviours among non-human primates’.

## 1. Introduction

Among primates, information about actual or potential social partners is valuable. For humans, each of us carries complex models of our social partners' personality, relationships and history in our minds, and most of us will expend considerable time and effort to exchange what we know about third parties with our immediate social partners; put more simply, we gossip. In industrialized societies, the economic value of this social information is especially clear for the parasocial relationships [1] that many people form with public figures, with a large and profitable industry selling social information in the form of gossip magazines, celebrity biographies, reality television shows, and so on. Non-human primates also have sophisticated mental models of their social partners, which include third-party social relationships [2]. Likewise, laboratory studies have revealed that social information carries economic value for monkeys, with animals foregoing the opportunity to consume desirable juice in order to consume information about social partners [3]. In more natural contexts, however, both human and non-human primates must often make decisions about which individuals to track, what behaviours are meaningful, and how best to allocate their limited time for information gathering to acquire the most valuable social information possible. Understanding how primates solve this complex problem requires combining and extending our best mechanistic models for decision making.

The framework of economic choice has in recent decades provided powerful insights into neural mechanisms underlying how humans and other animals solve the problem of selecting one target of consumption from a rich set of options. Several researchers have synthesized these findings into a ‘neuroeconomic' model of the decision process [4–6], which proposes that the brain first binds the perceptual features of available goods to positive and negative valence, a ‘top-down' valuation process that integrates the subjective weighting of those features along with the costs of the actions required to obtain the goods into a single ‘common-currency' representation. The brain then performs a comparison operation between these representations, selects the good with the highest economic value and then engages in the action to consume that good. It has been proposed that in humans and other primates, the orbitofrontal cortex (OFC) converts perceptual information, stored information about prior outcomes and internal state [7] into a single common-currency representation of the goods on offer [4–6], while the lateral prefrontal cortex (LPFC) serves to translate value represented in this ‘goods-space' to an ‘action-space’ of behaviour required to obtain the good [6,8].

A second, ‘state-space’ computational framework posits that the brain extracts, represents and applies information about hidden structure in the environment to guide decisions. This approach hypothesizes that humans and other animals model the environment as a state-space, using information about the current state of the environment and the organism within it to guide decisions and then updates the state-space representation as new information becomes available. Flexible behaviour such as maze navigation and reversal learning in both humans and rodents implicates OFC in representing the state-space, a view closely linked to the concept of a cognitive map [9–12]. In this view, the observation that OFC neurons signal the subjective value of goods on offer may reflect its more general-purpose role in tracking the state of the environment for making decisions. If this is correct, OFC is well-situated to carry representations of the social environment necessary for understanding and predicting the behaviour of conspecifics as well. While speculative, this idea is partially supported by the observation that a higher proportion of neurons in OFC encodes social category than fluid reward amount in an economic choice task, even when animals' decisions are primarily driven by the fluid reward [13].

The last two decades have witnessed a renewed interest in ethological approaches to understanding how the brain generates adaptive behaviour. This ‘neuroethological approach’ posits that the mechanisms supporting decision making evolved within, and are shaped by, the ecological and social contexts confronted by an organism during its lifetime [14,15]. While on the one hand, this approach emphasizes the importance of considering more naturalistic, domain-specific decision problems, it also motivates the use of tasks and stimuli in studying decision making that more accurately mimic the richness and complexity of the natural environment. The neuroethological approach has been applied to foraging decisions [16–18] and social decisions [3,13] in non-human primates—two of the most important and complex decisions primates make, and which are thought to be key drivers in the expansion of the neocortex during primate evolution [19–21].

The neuroethological framework emphasizes the challenges posed by the physical and social environment during both evolution and individual development. This assumes that animals learn both the hidden structure of the environment, as posited by the state-space model, and the economic value of available options for guiding behavioural decisions. However, these functions may not be cleaved neatly within the brain [14,15]. This contention is supported by a prior study by Watson and Platt [13] that studied how OFC neurons encoded information in macaques choosing between fluid rewards and the opportunity to obtain visual information about other monkeys, which has intrinsic adaptive value for guiding behaviour. The authors found that OFC neurons signalled information about both the type and subjective value of social images monkeys chose to view.

Consistent with these findings, Sliwa & Friewald [22] recently identified a ‘social interaction network' (SIN) in the macaque brain that is selectively engaged during the observation of social interactions but not interactions with objects. This social interaction network included multiple areas in the frontal cortex, including the OFC. The spatial and temporal limitations of functional magnetic resonance brain imaging (fMRI), the technique used by Sliwa and Friewald to uncover the SIN, leave unanswered whether individual neurons in OFC purely encode information about social interactions, their behavioural value, or both.

**Figure 1.**
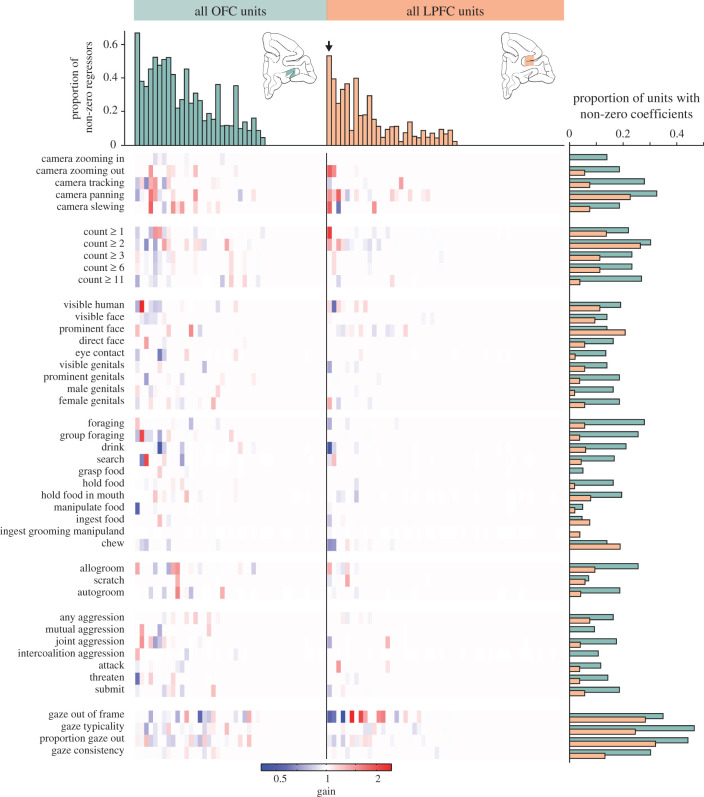
Modelled effects of ethogram and gaze variables on neuronal firing rates for all units in OFC (*n* = 56) and LPFC (*n* = 63). Each column represents one unit, and each row a regressor. The effect of each variable is encoded on the colour axis. Arrow indicates LPFC unit shown in figure 7*b,c*. Representation of ethogram and gaze variables in the population of PFC units. Histogram on the right shows the number of units with non-zero coefficients were counted and represented as a proportion of units in the OFC (green) or LPFC (orange) for each regressor. Histograms on the top show the proportion of regressors (out of 44) for each neuron in the OFC (green) or LPFC (orange).

**Figure 2.**
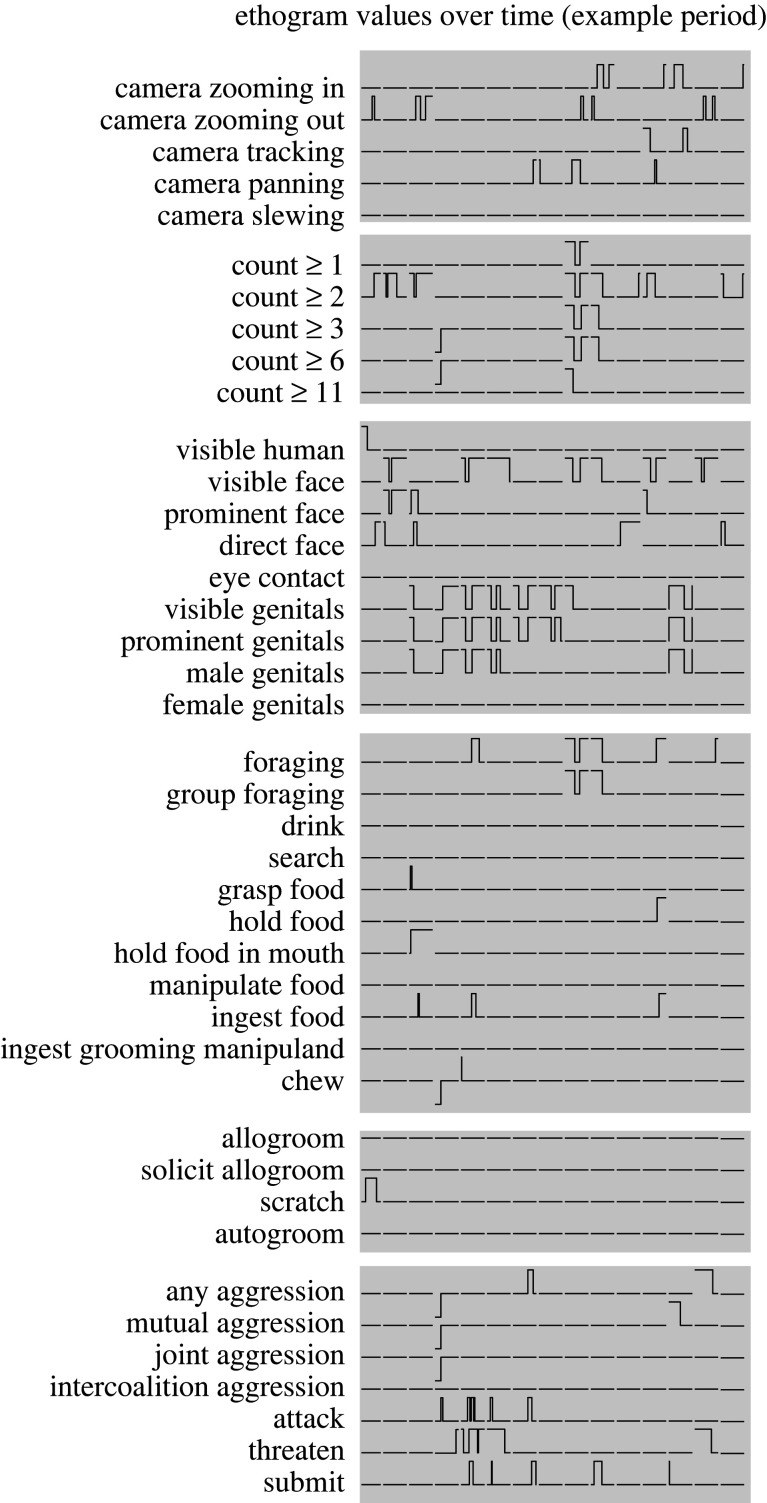
Schematic representation of ethogram variables during sequential free-viewing (FV) periods from a selected section of one behavioural session. Each horizontal row represents the value of one ethogram variable over time, and each vertical column of traces represents one 5 s FV period.

Together, these findings invite the possibility that more naturalistic task environments presenting ecologically relevant stimuli and eliciting species-typical behaviours may unmask the native coding scheme used by OFC, and that this area may be particularly engaged to learn about the structure of the current social environment. Here, we test this idea by exploring how rhesus macaques value the opportunity to acquire information about a richer, more naturalistic social environment and then how they spontaneously explore it. Each trial began with a two-alternative forced-choice (2AFC) task in which monkeys chose between two options selected randomly from a set of four on each trial: (i) blank screen; (ii) watch 5 s of a randomly selected video; (iii) re-watch the same 5 s video viewed on the last trial; (iv) watch the next 5 s following the video watched on the previous trial. During the video presentation, monkeys were free to look wherever they chose on or off the screen (free-viewing, FV). Videos were selected from a large corpus of videos of unfamiliar rhesus macaques engaging in a wide range of natural behaviours in a naturalistic setting, which had been annotated frame-by-frame using an established ethogram. This approach yielded multiple measures of behaviour: first, the measure of ordinal preferences among both the type and content of visual information, from which we could infer their economic value; second, the unconstrained spontaneous gaze behaviour monkeys used to explore the videos, which best simulates the rich environments in which they have evolved; and third, the study of single-cell neural activity during the exploration of this naturalistic environment, which allows us to discern the precise coding of OFC and LPFC neurons. We found that OFC neurons, as well as neurons in LPFC, encoded information about both the content of videos (figure 1) and the option identity (table 1 and figure 2), which indicates the ability of these neurons to address both short and long-term decisions (during free-viewing and while choosing of video clips), depending on the task at hand. Notably, while both brain regions responded to both social information and choice presentation, the social category was more richly represented in OFC, while more units in LPFC responded to and discriminated choice targets, a finding which is broadly consistent with the ‘goods-based' model of decision processing in these areas [5,6,8]. These findings endorse the hypothesis that OFC and LPFC integrate information about relevant objects and events, as well as their value—a representation suitable for both learning the hidden structure of the environment and guiding decisions within it.

**Table 1.** Responses of OFC and LPFC units to the 2AFC choice phase. Each neuron's spike count was modelled during choice presentation (0.1–0.6 s following target onset) with overdispersed Poisson generalized linear models (GLMs), with option types as predictors, and comparing to baseline firing rates (0.0–0.5 s prior to the start of each trial). Significance thresholds were Bonferroni corrected at 0.05.

		OFC (*n* = 56)		LPFC (*n* = 63)	
		% units significant	modulation (mean and range)	% units significant	modulation (mean and range)
chosen option model	target presentation	14.3% (8)	1.6 [0.26–18]	23.8% (15)	1.4 [0.28–4.1]
	continue (versus blank)	1.8% (1)	7.5	1.6% (1)	0.65
	switch (versus blank)	5.4% (3)	1.0 [0.33–2.2]	9.5% (6)	0.68 [0.28–1.4]
offered option model	target presentation	18.7% (10)	1.4 [0.057–17]	28.6% (18)	1.3 [0.21–8.8]
	continue (versus blank)	0% (0)	N/A	0% (0)	N/A
	switch (versus blank)	5.4% (3)	0.9 [0.42–1.5]	11% (7)	1.0 [0.45–2.2]

## 2. Methods

### (a) General methods and animal preparation

All procedures were approved by the Duke University Institutional Animal Care and Use Committee and were designed and conducted in compliance with the Public Health Service's Guide for the Care and Use of Laboratory Animals. Three adult male rhesus macaques (*Macaca mulatta*) participated in this study.

Prior to beginning the study, a small prosthesis for head restraint was implanted in each monkey using standard sterile surgical techniques. Following a six-week recovery period, the monkeys were trained to perform visual-oculomotor tasks for fluid rewards [23]. A second surgery was then performed to place a Cilux plastic recording chamber (Crist Instruments, Hagerstown, MD) above the prefrontal cortex. Monkeys received analgesics and antibiotics during recovery from both surgeries. After implantation, the recording chamber was routinely treated with antiseptic flushes, including before and after each recording, and between recordings was kept sealed with sterile caps.

During an experimental session, a monkey sat in a primate chair (Crist Instruments, Hagerstown, MD) in a darkened room facing a computer monitor. Stimuli were displayed on the monitor under the control of custom scripts programmed in Matlab (The Mathworks, Natick, MA) with the Psychophysics Toolbox [21]. Eye position was optically tracked at 1000 Hz via real-time pupil detection using an Eyelink 1000 system (SR Research, Mississauga, ON, Canada). Monkeys interacted with the task control system by aligning gaze with stimuli on the monitor. Juice rewards were delivered to a tube placed at the monkeys' lips from a reservoir by opening a solenoid valve, or by a peristaltic pump.

### (b) Behavioural techniques

#### (i) Video selection task

**Figure 3.**
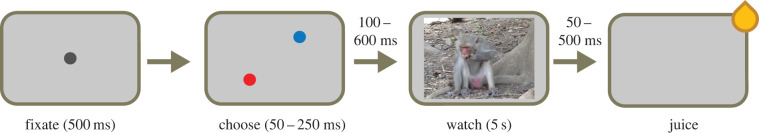
A schematic of the ‘channel surfing' task, in which 2AFC decisions were interleaved with free-viewing periods.

The task consisted of a series of trials, each composed of a fixation phase, a 2AFC phase, a 5 s free-viewing (FV) phase and a reward phase (figure 3). Monkeys initiated each trial by aligning gaze with a black disc (±0.5 deg diameter) positioned centrally on the computer monitor against a grey background for 500 ms. After maintaining gaze on the fixation target for 500 ms, the fixation target was removed, and either two (90% of trials) or one (10% of trials) eccentric coloured choice targets were presented. Monkeys selected one of the targets by aligning gaze with it (±4 deg) for 50–250 ms. Target colour indicated what type of video would play during FV if selected. There was a total of four types of 2AFC targets with associated outcomes: Blank (a static grey screen), Switch (a video clip randomly chosen from the library, weighted by its length and a start time was randomly selected within the file), Continue (continuation of the previous trial's video clip for the next 5 s in the video sequence) and Repeat (repetition of the previous trial's video clip). On each trial, the targets presented (the ‘menu') were randomly selected from these options. If there was less than 5 s of video available to continue from the previous video, ‘Continue' was not presented. If ‘Blank' was chosen, ‘Continue' and ‘Repeat' were never presented in the next trial, as these were not valid follow-ups to the static grey screen. In some sessions, ‘Repeat' was not included in the set of options. Upon registration of the monkey's choice, there was a brief (randomized between 100 and 600 ms) delay, followed by video playback for 5 s as determined by the selected target. Following video playback, there was a brief (randomized between 50 and 500 ms) delay, and then monkeys were rewarded with a drop of juice for successfully completing the trial. Juice rewards did not depend on monkeys' viewing decisions or gaze behaviour.

To ensure that ‘Continue’ and ‘Repeat' were presented at similar frequencies to ‘Switch' and ‘Blank', an adaptive weighting procedure was used when randomizing the menu for each trial. This was necessary, since these options could not be presented following a trial in which ‘Blank' was chosen, and ‘Continue' could not be presented if the previous video clip occurred at the end of the file from which it was drawn. Each possible menu (pair of options, or single option for forced-choice trials) was assigned a target weight *b_k_*, with forced-choice weights making up 10% of the total, and the target weighting otherwise uniformly distributed and summing to 1. Throughout the task, the number of times each menu option had actually been presented *n_k_* out of the total number of trials so far *N* was tracked and used to calculate the per-trial adaptive weight *a_k_* with the following formula:

$$a_{k}=b_{k}2^{(1-n_{k}/(Nb_{k}))/0.2}.$$

This function was selected empirically during early development of the task paradigm to avoid overly large changes in the probability of seeing any given menu type on the basis of recently presented menus, with the constant 0.2 being a ‘reweighting rate' that was fixed to provide the desired behaviour.

A video stimulus set consisting of 4.87 h of the video in 429 files was recorded in August 2009 at the Caribbean Primate Research Center's Cayo Santiago facility in Puerto Rico. The footage was recorded with the goal of representing a wide range of natural behaviours. Videos included no cinematographic edits (e.g. cuts), but did include camera movements and changes in zoom level, in order to both capture behaviours of interest and to allow the videographer to move in response to potentially aggressive monkeys.

For compatibility with the experimental control software, all video files were converted to Apple Quicktime format at 610 width by 458 height resolution and 30 frames per second. Because the sound track's quality was highly variable, and frequently included vocalizations from animals out of frame which were unrelated to the visible events, the audio was removed from all videos.

### (c) Neurophysiological techniques

We recorded action potentials from 63 single units from the LPFC in the principal sulcus (monkey C: 31, monkey E: 32) and 56 single units from the OFC (monkey C: 29, monkey E: 27). Recordings were performed using either single-wire tungsten microelectrodes (Fred Haer Co., Bowdoin, ME) or an 8-channel multi-contact probe (U-Probe, Plexon Inc., Dallas, TX) configured with two tetrode clusters. During recordings, the probes were guided to the intended recording site using a hydraulic microdrive (David Kopf Instruments, Tujunga, CA). Target recording sites were determined through the conjunction of several techniques. All recordings were performed using a standard recording grid (Crist Instruments, Hagerstown, MD). Structural MRIs were obtained for monkeys E and C, and the distances between the dorsal surface of the brain below each grid hole and the intended recording sites were measured. Observations of changes in audible broad-band power and multi-unit activity electrophysiological landmarks during the positioning of the electrode prior to recording (as determined by sending the amplified electrophysiological signal to a speaker) were used to further confirm the recording site. Finally, additional anatomical information was obtained via ultrasound imaging through the recording chamber [24] using a hand-held digital ultrasound device (SonoSite 180, FUJIFILM SonoSite, Inc.).

### (d) Analytical techniques

#### (i) Ethogram and video annotation

We annotated stimulus videos using an ethogram of rhesus macaque behaviours [25] but adapted for the constraints of the videos and the requirements of our study. Videos were manually scored with a custom program (Tinbergen Alpha; [26]), producing a complete timeseries of observable behavioural events for each video. In addition to behaviours, additional descriptors of the video scenes were scored and included alongside behavioural data. These included camera movements and changes in zoom level, the number of monkeys present in the scene, and descriptors of the visibility of the face and ano-genital regions (two highly salient sources of social information for macaques—see [3]). For simplicity, we will refer to the complete scoring scheme, including both behaviours and view-related events, as the ‘ethogram', a mild stretch of terminology reflecting our focus on describing observable stimuli and behaviour in the videos.

We found while scoring and analysing our data that the ethogram needed to be structured slightly differently for these two purposes. In particular, scoring was facilitated by treating certain behaviours as states that could occur at several levels, while analysis was facilitated by treating every behaviour as a binary variable. Additionally, after scoring we discovered that some specific behaviours occurred too infrequently to analyse, but could be pooled with other behaviours into a natural class with a broader definition. Table 2 provides the complete ethogram used for scoring, and table 3 provides the ethogram used for analysis, data for which was derived from the scoring ethogram. By combining the ethogram data with the record of which video was presented during each FV phase, a record of the behaviours viewed by a monkey over time within a session could be produced and used as a regressor for behavioural and neurophysiological analysis (figure 2).

**Table 2.** Scoring ethogram.

observation	levels	description
camera movement	holding	the camera is not moving much, holding a mostly stable image
	zooming in	the camera is holding a mostly stable image, but is zooming in
	zooming out	the camera is holding a mostly stable image, but is zooming out
	tracking	the camera is tracking the movements of a monkey, holding it largely stable against the moving background
	panning	the camera is moving without tracking anything, and the scene is still viewable
	slewing	the camera is moving wildly and the scene is difficult to view
count	0	the number of monkeys visible in the scene, scored in approximately logarithmically spaced levels
	1	
	2	
	3–5	
	6–10	
	>10	
human visible		whether human researchers are visible in the scene
face visibility	none	no faces are visible with a view angle (the angle between the camera and the head axis) of less than *ca*. 100 deg.
	small	at least one face is visible with a view angle of less than *ca*. 100 deg., but none subtend a linear extent of greater than *ca*. 10% of the width of the video
	away	at least one face is visible with a view angle of between *ca*. 45 and 100 deg., and subtends a linear extent of greater than *ca*. 10% of the width of the video
	direct	as ‘away', but the view angle is less than *ca*. 45 deg.
	eye contact	as ‘direct’, but the monkey also appears to be looking directly at the camera
ano-genital area visibility	none	the ano-genital area (AGA) is defined as the glabrous, red skin around the genitals, anus and upper inner thigh (the ‘sex skin’), the scrotum on males, and the ischial callosities; the AGA of no monkeys is visible.
	small	at least one monkey's AGA is visible, but none are prominent on the screen
	unsexed	at least one monkey's AGA is prominently visible, but the observer is unable to sex the animal
	male	at least one male monkey's AGA is prominently visible
	female	at least one female monkey's AGA is prominently visible
	both	at least one male and one female monkeys’ AGA are visible
drink		any visible monkey is drinking water
forage	no	no monkeys are engaged in foraging behaviour.
	few	one or two monkeys are in a foraging state, characterized by various specific actions such as manipulating or ingesting food
	many	three or more monkeys are engaged in foraging behaviours; due to the rapid increase in complexity of coding specific actions with this number of foraging monkeys, specific actions are only coded during the ‘few’ condition
search		searching for a food item by manipulating foliage, ground substrate, or a pile of provisioned food items
grasp food		reaching for a food item; begins at initiation of the reach movement and ends once the food item is in hand
hold food		a food item is visibly held in hand or foot
hold food in mouth		a food item is visibly held in the mouth; carrying food in cheek pouches is not included
manipulate food		active manipulate of a food item other than grasping or ingesting, e.g. ‘washing’
ingest food		a food item is brought to the mouth: begins at initiation of arm movement and ends when the food item is consumed or the hand leaves the mouth area; includes movements of food item towards the mouth that do not actually result in consumption, e.g. ‘sniffing’
ingest grooming manipuland		a hand is brought from a grooming target (self or partner) to the mouth, as if the monkey is consuming a parasite plucked from the skin
chew		rhythmic jaw movements; an entire bout of chewing is coded rather than individual bites
retrieve from pouch		a food item is brought out from the cheek pouches
heave		a full body movement involving tightening of the abdomen and straightening of the esophagus, as though the monkey is preparing to vomit or in the act of vomiting
scratch		rhythmic, vigorous movement of the hand or foot against the monkey's own body
autogroom		self-directed grooming behaviour; involves more finely controlled hand and finger movements than scratching
allogroom		other-directed grooming behaviour; allogroom and autogroom involve similar motor actions, differing only in the target
solicit allogroom		a monkey approaches another monkey and sits or lies down, presenting itself for grooming
aggression level	none	no aggression is present in the scene
	unidirectional	a single individual is displaying aggressive behaviour
	bidirectional	two individuals are displaying aggressive behaviours toward each other
	joint	two or more individuals are jointly displaying aggressive behaviours, the target(s) of which may or may not be visible
	intercoalition	two ‘coalitions’ are displaying aggressive behaviours toward each other
strike		a monkey makes brief, aggressive physical contact with another, excluding shoving infants by adult females
grapple		two monkeys engage in prolonged aggressive physical contact
lunge		a monkey makes a short, aggressive movement toward another monkey
withdraw		a monkey backs away from another monkey while making aggressive or submissive displays
charge		a monkey makes a rapid, prolonged aggressive movement toward another monkey
flee		a monkey moves away from another monkey at high speed
chase		one monkey charges another monkey as it flees
threaten		a monkey performs a threat display, characterized by a round open mouth, prolonged staring, head bobbing, piloerection, and erect posture
mounted threaten		one monkey mounts another while both threaten a third monkey
submit		a monkey performs a submissive display, characterized by bearing teeth, squeaking and withdrawn posture
displace		one monkey walks directly toward or near another, which moves away
lean away		a monkey posturally shifts away from an approaching conspecific without fully withdrawing or displacing
avoid		a monkey pauses or alters course during movement to maintain greater distance to another monkey
branch display		a monkey vigorously shakes a branch or branch-like object (e.g. a metal pole)

**Table 3.** Analysis ethogram.

observation	definition
camera zooming in	‘camera movement’ is ‘zooming in’
camera zooming out	‘camera movement’ is ‘zooming out’
camera tracking	‘camera movement’ is ‘tracking’
camera panning	‘camera movement’ is ‘panning’
camera slewing	‘camera movement’ is ‘slewing’
count ≥ 1	‘count’ is ‘1’, ‘2’, ‘3–5’, ‘6–10’ or ‘>10’
count ≥ 12	‘count’ is ‘2’, ‘3–5’, ‘6–10’ or ‘>10’
count ≥ 13	‘count’ is ‘3–5’, ‘6–10’ or ‘>10’
count ≥ 16	‘count’ is ‘6–10’ or ‘>10’
count ≥ 111	‘count’ is ‘>10’
visible face	‘face visibility’ is ‘small’, ‘away’, ‘direct’ or ‘eye contact’
prominent face	‘face visibility’ is ‘away’, ‘direct’ or ‘eye contact’
direct face	‘face visibility’ is ‘direct’ or ‘eye contact’
eye contact	‘face visibility’ is ‘eye contact’
visible genitals	‘AGA visibility’ is ‘small’, ‘unsexed’, ‘male’, ‘female’ or ‘both’
prominent genitals	‘AGA visibility’ is ‘unsexed’, ‘male’, ‘female’ or ‘both’
male genitals	‘AGA visibility’ is ‘male’ or ‘both’
female genitals	‘AGA visibility’ is ‘female’ or ‘both’
foraging	‘forage’ is ‘few’ or ‘many’
group foraging	‘forage’ is ‘many’
any aggression	‘aggression level’ is ‘unidirectional’, ‘bidirectional’, ‘joint’ or ‘intercoalition’
mutual aggression	‘aggression level’ is ‘bidirectional’, ‘joint’ or ‘intercoalition’
joint aggression	‘aggression level’ is ‘joint’ or ‘intercoalition’
intercoalition aggression	‘aggression level’ is ‘intercoalition’
attack	any of ‘strike’, ‘grapple’, ‘charge’, ‘lunge’ or ‘chase’
threaten	any of ‘threaten’ ‘mounted threaten’ or ‘branch display’
submit	any of ‘withdraw’, ‘flee’, ‘chase’, ‘submit’, ‘displace’, ‘lean away’ or ‘avoid’

#### (ii) Modelling and analysis of two-alternative forced-choice decisions

To assess whether the observed behaviours during the FV phase had an effect on subsequent 2AFC decisions, we used a generalized linear model (GLM) with elastic net regularization [27], using the R packages ‘glmnet' [28] and ‘caret' [29]. Elastic net regularization is a technique that has been successfully applied in several areas of biostatistics and which has empirically been demonstrated to perform well with large numbers of correlated regressors [27] making it ideal for discovering which, if any, of the relatively large number of regressors taken from the ethogram might help predict monkeys' subsequent 2AFC viewing decisions (as well as neuronal activity—see below). The regularization parameter *λ_1SE_* and the elastic net mixing parameter *α* were identified for each unit via 20-fold cross-validation, such that *α* yielded the best fit model and *λ_1SE_* was the largest value for the regularization parameter yielding a model within one standard error of the best fit model [28].

We constructed the non-regularized GLM component of the model as follows. First, the full set of 2AFC menu options was assigned an arbitrary ranking: Blank = 0, Repeat = 1, Continue = 2, Switch = 3. (Note that the specific rank-ordering chosen had no impact on the final interpretation of the model.) For each 2AFC, the response variable was assigned 1 for selection of the higher ranked option and 0 for the lower ranked option. Next, for each of Repeat, Continue and Switch, a regressor was assigned 1 if it was the higher ranked option of the menu, −1 if it was the lower ranked option of the menu and 0 if it was not offered. Blank was treated as a baseline comparison condition and thus not assigned a regressor. Then, ethogram variables were assigned 1 if the entry occurred at all during the previous trial's video presentation, and 0 otherwise. 2AFC decisions were then modelled as Bernoulli trials, with the probability of the monkey choosing the higher ranked option given by the following equation:

2.1 $$\text{ln}\left(\frac{\;p_{d}}{1-p_{d}}\right)=\underset{i}{\sum }\upsilon_{i}m_{d,i}+\underset{i}{\sum }\underset{k}{\sum }\beta_{i,k}m_{d,i}x_{d,k}.$$

Here, *d* indexes the decision (or observation), *i* indexes the three menu regressors, *k* indexes the ethogram variables, *m_d,i_* is the menu regressor for option i, *x_d,k_* is the value of the *k*'th ethogram item, *v_i_* is a coefficient representing the overall utility of option *i*, and *β_i,k_* is a coefficient representing the change in utility of option *i* when ethogram item *k* was previously viewed (that is to say, an interaction term between an effect of the currently presented option and an ethogram entry in the previously viewed video). We then fitted the coefficients *v_i_* and *β_i,k_* to the data with a GLM under elastic net regularization, with *β_i,k_* subjected to the elastic net penalty while *v_i_* were not.

#### (iii) Measurement and analysis of gaze behaviour during free-viewing

Aggregating gaze behaviour observations across multiple FV sessions required transforming the optical eye position signals into a spatio-temporal coordinate system common to the presented videos rather than the one naturally defined by the spatial extent of the monitor and the time within the behavioural session. To interpret subject monkeys' gaze behaviour during FV, we temporally downsampled the 1000 Hz gaze signal to 30 Hz via boxcar averaging, minimizing the phase delay between the video frame drawing times and the centres of the boxcar windows, and spatially rescaled it to a normalized spatial coordinate system in which the height of the video was 1 unit and the centre of the video frame was the origin. We term the resulting signal *gaze focus*. Gaze focus was determined to be *in frame* when within the space defined by ±0.550 on the vertical axis and ±0.716 on the horizontal axis, a rectangle encompassing the video frame with a buffer on each side of 0.05 normalized units (i.e. 5% of the height of the video).

Videos of conspecifics frequently elicit characteristic gaze scan patterns in rhesus macaques [30–32]. Qualitatively, this was evident when observing gaze scan paths from the FV phase in our task as well. However, appropriately quantifying the ‘repeatability' of gaze behaviour, without explicitly referencing visual features within the scene, is a nontrivial problem. In particular, gaze focus is often strongly attracted to two or three features within a scene. This multimodality in the spatial distribution of gaze focus produces a very large covariance for the overall distribution, despite the fact that gaze focus may be tightly clustered around each mode. In fact, the eigenvalues of the covariance matrix are typically larger for this case than for scenes in which there are no strong attractors of gaze, and thus gaze focus is distributed randomly. Most standard approaches to assessing the variability of a distribution are directly or indirectly affected by this issue, and approaches based on directly modelling the multimodality of the gaze data (e.g. k-means clustering) proved to be impractical for this dataset due to long computation times. To address these issues, here we introduce a new metric which we term *gaze consistency*. For a moment in time with *n* gaze focus observations, gaze consistency is defined as

2.2 $$g=\frac{2d_{0}}{n(n-1)}\underset{i}{\sum }\underset{\;j<i}{\sum }\frac{1}{\text{max}(∥x_{i}-x_{j}∥,\;d_{0})},$$

where *x_i_* and *x_j_* represent the position vectors for pairs of gaze focus observations, and *d_0_* is a constant estimating the smallest distance between two points considered to represent a meaningfully different focus of gaze. For analysis, we defined *d_0_* as 0.01, that is, 1% of the height of the video, 4.58 video pixels, or approximately 0.23 degrees of visual arc.

Gaze consistency is mathematically analogous to (and inspired by) the potential energy of a collection of particles following a repulsive inverse-square law (e.g. a cloud of classical electrons), with each ‘particle’ representing the focus of a monkey's gaze in a particular video frame from a single viewing. (It differs from this physical analogy in that the ‘force' between ‘particles' is limited by the parameter *d_0_*.) It has a theoretical maximum value of 1 if all observations of gaze were to fall within a disc of diameter *d_0_*, and decreases toward zero with the greater disparity in the focus of gaze upon repeated viewings of the same video frame. Importantly, gaze consistency is only moderately sensitive to the presence of multiple clusters of gaze focus. A single tight cluster of gaze focus yields a greater consistency value than two such clusters, but two clusters yield a greater gaze consistency than a diffuse ‘cloud' of gaze, a relationship that does not hold for, e.g. the covariance of gaze focus. Thus, gaze consistency values tend to track, in an ordinal fashion, the qualitative sense of ‘clustered-ness' in the gaze focus observations.

In addition to gaze consistency, we also employed a related metric we term *gaze typicality*, which indexes how similar an individual observation of gaze focus is to other observations for the same video frame, defined as

2.3 $$\tau_{i}=\frac{d_{0}}{n-1}\underset{\;j\neq i}{\sum }\frac{1}{\text{max}(∥x_{i}-x_{j}∥,\;d_{0})}.$$

Using the same physical analogy as for gaze consistency, this metric is analogous to the potential energy of a single particle within the cloud of particles. Like gaze consistency, gaze typicality theoretically ranges between zero (highly atypical gaze) and one (highly typical gaze). The gaze typicality for a particular observation of gaze focus is high when there are many similar such observations for that frame, and low when it is distant from other observations of gaze focus.

Conceptually, gaze consistency is a property associated with a video frame, and periods of high gaze consistency indicate video sequences that present features or events which are potent attractors of gaze, even without necessarily knowing the nature of those features or events. By contrast, gaze typicality is a property associated with an individual gaze trace and indicates the extent to which the animal's gaze during a particular video presentation was attracted to the regions which, on average, tend to attract gaze. Gaze consistency and typicality are useful metrics for the interpretation of gaze behaviour in a large, diverse stimulus set such as the one we employed for this task, offering the advantages of being invariant to the position (i.e. it does not matter where in the visual field a feature that attracts gaze may be) and simple to calculate. However, it is important to interpret these values carefully, as they are highly sensitive to the relative size of features in the visual field (or in other words, they are not scale-invariant). In particular, a scene containing a feature such as a face, which is a potent attractor of gaze but spans a large region of the visual field, will tend to yield lower gaze consistency than a scene which contains a similar feature which spans a smaller region of the visual field.

Gaze consistency and gaze typicality were calculated only for frames with at least five in-frame observations of gaze focus, and gaze typicality was additionally only calculated for in-frame gaze focus. In addition to these metrics, we also examined whether each gaze focus was in frame (*gaze onscreen*) and the overall proportion of in-frame gaze focus for all viewings of each frame (*proportion gaze onscreen*).

#### (iv) Analysis of neurophysiological data

To investigate the relationship between viewed behaviours and neuronal firing rates during the video presentation, we modelled neuronal spike counts as following a Poisson distribution with parameter $\phi_{n,t}$, where *n* indexed distinct video presentations and *t* the time within each presentation. This rate parameter depended on an intrinsic baseline rate modulated by a set of independent variables taken from the ethogram data and monkeys' gaze behaviour, as follows:

2.4 $$\text{ln}(\phi_{n,t})=ln(\phi_{0}\pi_{t}\delta_{n})+\underset{k}{\sum }\beta_{k}x_{k,n,t-\tau }.$$

**Figure 4.**
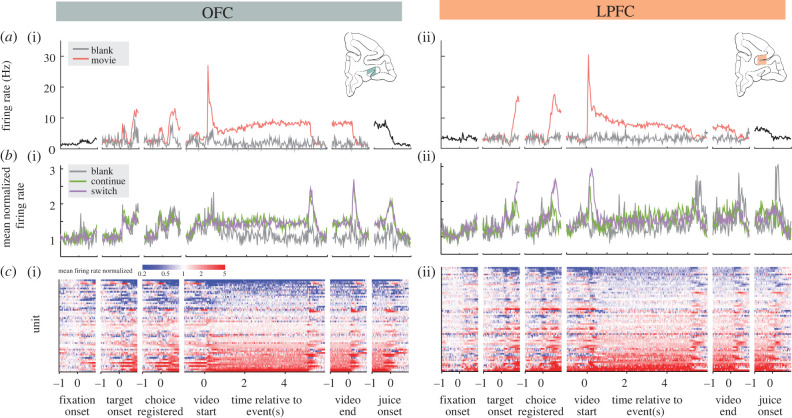
(*a*) Single-unit firing rate responses for example units in OFC (i) and LPFC (ii). Each trace represents the mean normalized firing rate for the neuron when a movie (red) or the blank grey background (grey) was presented. (*b*) Population-averaged firing rate responses to trial events in OFC (i) and LPFC (ii). For each unit, firing rates were normalized to the 500 ms prior to fixation onset. Each trace represents the mean normalized firing rate across the population for those trials in which the monkey selected ‘Blank' (grey), ‘Continue' (green) or ‘Switch' (purple). Because the option ‘Repeat' was included only for a subset of units, it is omitted from the data shown. (c) OFC (i, *n* = 56) and LPFC (ii, *n* = 63) units' responses to task events were heterogeneous. Each horizontal row represents one individual unit's peri-event activity, and the colour of each cell indicates the normalized firing rate during a time bin. Units are ordered within each region by mean normalized firing rate during the video presentation epoch.

Here, $\phi_{0}$ is a baseline firing rate for the cell, $\pi_{t}$ is a characteristic timecourse of activity for the cell during video playback, calculated from the conventional peri-stimulus time histogram across all video presentations for the unit, $\delta_{n}$ is a per-trial ‘drift' accounting for the fact that some units changed their baseline activity substantially over several hours of recording, $\beta_{k}$ is a ‘gain' factor describing the effect of each independent variable on the firing rate of the unit (found by fitting the model to our observations via GLM) and $x_{k,n,t-\tau }$ is the value of the *k*'th independent variable in the *n*'th trial at a time *t* – *τ*. The value *τ* is an estimate of the latency for processed visual information to arrive in the prefrontal cortex and was set as 100 ms on the basis of the typical delay between the onset of video playback and the change in activity that was observed in most of our units (figure 4).

Because some behaviours in the ethogram occurred relatively infrequently, it was possible for our observations of neural firing to include only a small number of presentations involving these behaviours. This was of concern not only because of the small sample size, but also because with only a small number of exemplars of a particular behaviour, it is impossible to determine whether changes in neural firing rate were due to the behaviour itself or an unrelated event that happened to co-occur with the behaviour in one or two of the presentations. Therefore, for each item in the ethogram, we required that it be present in at least five distinct presentations for its regressor to be included in the model as an independent variable explaining the firing rate.

We were also interested in how PFC neurons' firing rates related to monkeys' gaze behaviour. As estimators of intrinsic elements of the video scene that influenced monkeys' gaze, we included gaze consistency and the proportion of gaze-out-of-frame observations as regressors along with the ethogram variables. As estimators of per-view decision processes, we included gaze typicality and gaze-out-of-frame as regressors as well.

A final challenge was the principled selection of the window duration used to count spikes, each window forming an independent observation in our regression model. Because both neuronal firing rates and ethogram regressors are temporally autocorrelated, selection of an analysis window shorter than the scale of neural firing rate autocorrelation would bias our model toward false positives, due to the fact that adjacent bins would be highly correlated in the data while treated as independent by the model. Conversely, the selection of too large an analysis window would risk failing to adequately represent some of the briefer behaviours in the ethogram and could violate the assumption of stationarity within each window. The autocorrelation function, for example neurons taken from the 5 s window during Blank presentations, revealed that the autocorrelation coefficient falls to below 0.1 at a lag of approximately 100 ms in the absence of task stimuli (electronic supplementary material, figure S1). On this basis, we selected a 200 ms window (twice the 100 ms cutoff) to balance the needs to avoid autocorrelation and maintain stationarity within each window. Thus, each video presentation was divided into twenty-five 200 ms observations of neuronal firing rate, each of which could be considered roughly independent for the purposes of statistical analysis. Because the observation window was larger than a single 33 1/3 ms video frame, ethogram values were averaged over the window to compute the regressors. We then fitted the coefficients *β* to the data using a logistic GLM with elastic net regularization.

## 3. Results

### (a) Monkeys preferred new information over old information or blank screens, but video choices did not reflect video content

**Figure 5.**
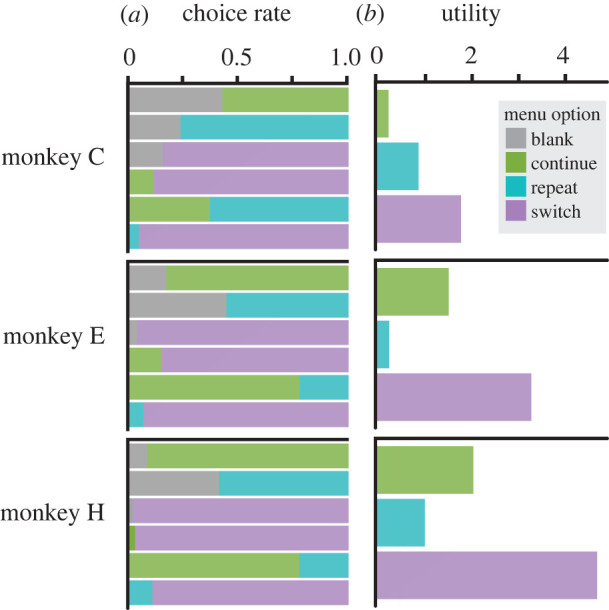
(*a*) Each monkey exhibited clear preferences among viewing outcomes. Each vertical pair of bars represents one ‘menu' of paired options presented in the 2AFC phase of the task. The relative height of each bar indicates the proportion of decisions for the associated option when presented against the opposing option in the menu. (Monkey C: 42,229 decisions, monkey E: 29,951 decisions, monkey H: 14,713 decisions.) (*b*) The associated utility for each option for each monkey, fit by logistic GLM. Utility values are relative to the ‘Blank' option.

All three monkeys displayed marked preferences among the four menu options (figure 5*a*). Notably, for all three monkeys, the least preferred option was Blank, and the most preferred was Switch, indicating a general preference for video stimuli over blank screens, and for unpredictable video stimuli over stimuli predictable from the previous trial's presentation. Contrary to our original expectations, our model of 2AFC decisions failed to discover any effect of viewed behaviours on subsequent choices, yielding no non-zero coefficients for the option-ethogram interaction terms for any of the subject monkeys. However, the model did allow us to estimate the main effects of option identity, which we interpret as the economic value of each type of video (figure 5*b*).

### (b) Gaze behaviour strongly varied with video content

**Figure 6.**
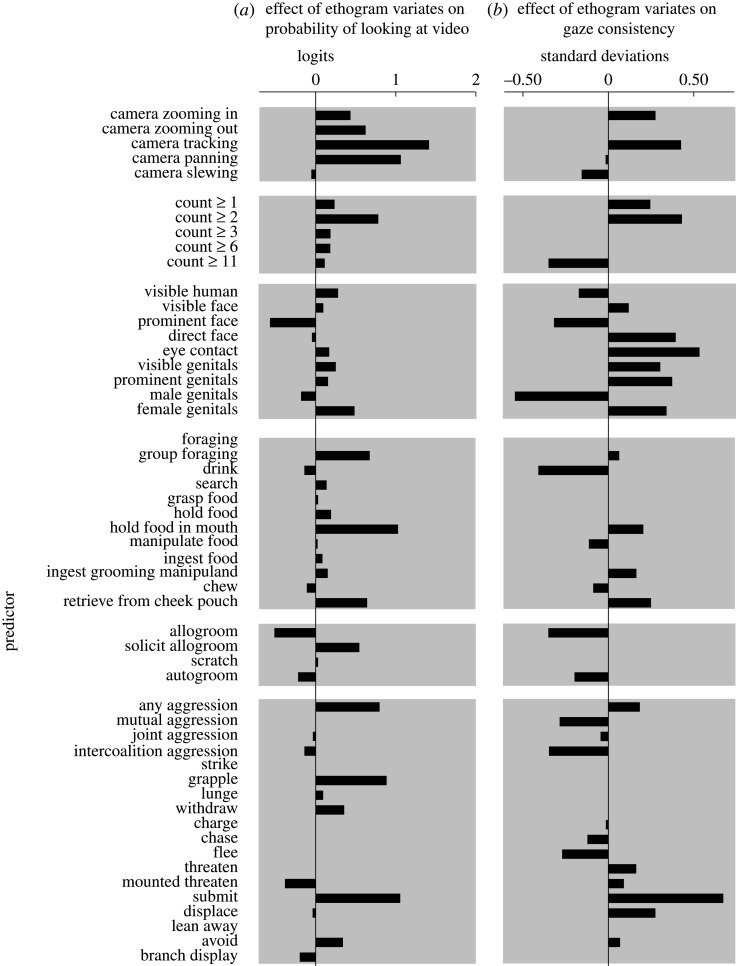
Observable behaviours by conspecifics influenced monkeys decisions to look at or away from the video during free-viewing. (*b*) Conspecific behaviours also influenced the consistency of monkeys' gaze behaviour during free-viewing.

Gaze focus data was aggregated across all repeated viewings of each given video sequence (for an example, see electronic supplementary material Movie). To assess how monkeys' gaze behaviour varied with stimuli and events in the videos, we modelled both *gaze consistency* and *gaze onscreen* as dependent variables in GLMs with elastic net regularization, as described above. Each presentation of a video frame represented an observation (12.6 million total video frame presentations), with the associated ethogram variables as independent regressors. Gaze onscreen was modelled with the logit link function (binomial distribution), and gaze consistency was normalized and modelled with the identity link function (normal distribution). Unlike the case for monkeys' explicit 2AFC decisions, we found that many regressors had a non-zero effect on both the decision to look at the video frame and on gaze consistency within the videos (figure 6).

Of particular note, camera movements in general seemed to enhance the visual interest of the video, with the exception of the wild ‘slewing' camera movements during which individual features in the video were difficult or impossible to distinguish. ‘Tracking' camera movements, in which the camera followed a moving animal to stabilize it in the frame, were particularly effective at attracting gaze to the scene. Monkeys' probability of looking at the video also increased with increasing numbers of conspecifics visible, with a particularly large increase associated with the second visible monkey (count ≥2). By contrast, and perhaps somewhat counterintuitively, the prominent visibility of a face (face prominent) decreased the probability of looking at the video. This effect is consistent, however, with previous findings that the utility of social information may be decoupled from looking-time in rhesus macaques for faces [3], perhaps because direct gaze can be a threatening behaviour in this species [33].

In addition to these purely visual features, several observable behaviours significantly influenced the decision to look at the video. Aggressive behaviour (any aggression) increased the probability of looking at the video, with prolonged aggressive physical contact (grapple) being particularly effective at attracting gaze to the scene. Submissive displays (submit) were also effective attractors of gaze to the overall scene. Among the foraging behaviours, ‘group foraging', ‘hold food in mouth' and ‘retrieve from cheek pouch' were particularly effective at attracting gaze to the video, consistent with the phenomenon of vicarious reinforcement [34]. The first of these likely reflects the high information density when numerous conspecifics are behaving in the scene, and the latter two effects possibly indicate that close proximity of a food item to a conspecific's mouth is highly salient for rhesus macaques. Somewhat surprisingly, ‘allogroom' was associated with a reduction in the probability of looking at the video, despite its well-established importance for establishing and maintaining social relationships in macaques [35]. We speculate that because allogrooming is typically a lengthy behaviour during which novel information is unlikely after the initial observation, the marginal utility of continued observation is low, decreasing the utility of continuous attention to the behaviour for an external observer.

Like monkeys' decisions to look within from the video frame, gaze consistency was modulated by both visual features and viewed behaviours, but the nature of this metric (and in particular the fact that it is not scale-invariant) means that the coefficients discovered by our model must be interpreted with some care. For example, direct, non-quantitative observations of the original videos with pooled observations of gaze focus (electronic supplementary material, video) make it apparent that monkeys tend to consistently direct gaze towards male genitals when they were present, yet the model reports a low-gaze consistency associated with periods when male genitals are visible on the screen. Although it should be noted that because ‘male genitals’ is a nested regressor within ‘prominent genitals' and ‘visible genitals’, the total gaze consistency prediction for periods when male genitals are visible on screen is therefore the sum of these three values. Thus, the comparatively large negative value for ‘male genitals' indicates low-gaze consistency relative to what would otherwise be expected for prominently visible genitals. Due to the size of the rhesus scrotum, the adult male ano-genital area (AGA) is comparatively larger and visible at a wider range of viewing angles than the perinea of females or juveniles (juveniles comprising the majority of the ‘unsexed' prominently visible AGAs in the video database). This larger ‘natural scale' means that gaze foci within the male AGA are more likely to be widely separated compared to multiple gaze foci within a non-male AGA, hence the negative effect of the factor ‘male genitals' on gaze consistency. The result of the model for this feature highlights a limitation of gaze consistency as a metric: an effect of the stimulus on gaze behaviour can manifest as either a positive or negative change in gaze consistency, and the magnitude of change in gaze consistency cannot necessarily be compared between different types of stimuli.

Despite this caveat, the model fitted to the gaze consistency data reveals a number of intriguing patterns. As with gaze onscreen, gaze consistency decreased with the presence of a prominently visible face in the scene. However, unlike gaze onscreen, which showed little change as the view of a face moved from prominent, to direct, to making eye contact with the camera, the trend for gaze consistency reversed, rising with these increasingly ‘intense' views of a face. This finding is consistent with previous reports that monkeys' scan patterns of faces tend to be highly stereotyped. Also notable is the comparatively large increase in gaze consistency associated with ‘submit', which parallels the increase in gaze onscreen probability, suggesting that submissive displays were of great visual interest. By comparison, the increase in gaze consistency was much more modest for ‘threaten’, and no effect was found for this behaviour on gaze onscreen. We speculate that this difference may exist because while both of these behaviours are highly informative social signals, monkeys may have been more prone to quickly avert their gaze from threat displays [36], as under natural conditions prolonged eye contact with a threatening conspecific could provoke an attack.

### (c) Neurons in orbitofrontal cortex and lateral prefrontal cortex encode both social information and economic value

We recorded from 119 individual neurons in both OFC (*n* = 56) and LPFC (*n* = 63). By analysing neural activity during both the 2AFC phase and the FV phase, we aimed to understand how well current theories of this system map onto decision making in a complex, naturalistic context. Population-average peri-event time histograms (PSTHs) revealed that both OFC and LPFC responded to the major events within the task's trial structure, with some individual neurons in both areas showing particularly strong and sustained activation during video playback (figure 4). Spike counts during 2AFC (100–600 ms following target presentation) were modelled with overdispersed Poisson GLMs, using either the identity of the chosen option or of the offered options as predictors. In both OFC and LPFC, a modest fraction of units responded to the presentation of targets (chosen option model: OFC, 8 [14.3%]; LPFC, 15 [23.8%]/offered option model: OFC, 10 [18.7%]; LPFC, 18 [28.6%]), but only a small proportion of units further discriminated the Switch option (chosen option model: OFC, 3 [5.4%]; LPFC, 6 [9.5%]/offered option model: OFC, 3 [5.4%]; LPFC, 7 [11%]) or the Continue option (chosen option model: OFC, 1 [1.8%]; LPFC 1 [1.6%]/offered option model OFC, 0; LPFC, 0) despite monkeys' strong ordinal preferences.

LPFC, as a whole, responded vigorously and phasically to the onset of videos, but not to the blank screen condition (which involved no change in the visual stimulus). OFC, by contrast, appeared to show a much greater overall increase in activity throughout video presentation and a marked phasic response to the end of the video (at which time the screen returned to a neutral grey colour). Notably, LPFC exhibited a phasic response to the delivery of juice following blank presentations, but not to juice delivery following videos, possibly reflecting the fact that the end of the video playback was a reliable predictor of upcoming juice delivery, whereas no similar visual cue occurred at the end of the blank screen ‘playback' period. Also of note, within both LPFC and OFC, many neurons showed activation patterns that deviated from the population averages (figure 4*c*), suggesting that there was considerably more information contained within the activity of these ensembles as a whole than is reflected in the population mean.

**Figure 7.**
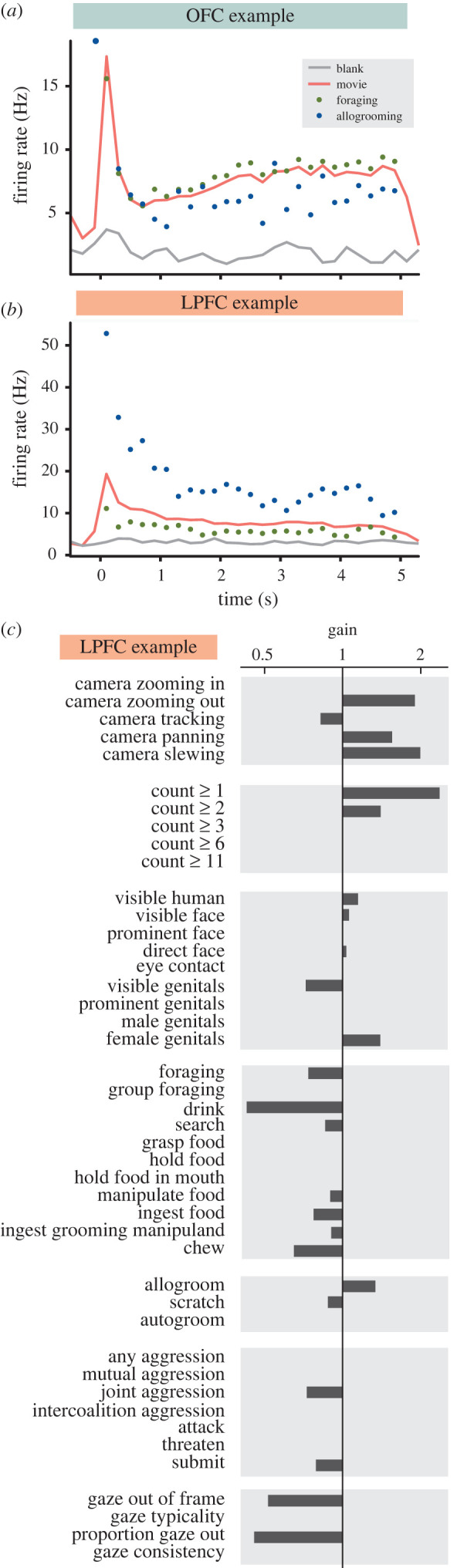
PFC units' firing rates were modulated by viewed behaviours during free-viewing. (*a,b*) Example units from LPFC (*a*) and OFC (*b*). Connected lines represent PSTHs during presentation of a movie (red) or blank screen (grey), with 200 ms bins. Unconnected points represent mean firing rates for each bin across only those presentations in which foraging (green) or allogrooming (blue) was occurring. Because behavioural events did not necessarily span the entirety of a 5 s video presentation, each point may represent bins from a different subset of all FV periods for that unit. (*c*) Modelled effects of ethogram and gaze variables on neuronal firing rate for the unit shown in (*b*). The horizontal axis (gain) is logarithmically spaced.

To understand whether the content of the FV scenes modulated the activity of individual neurons, we compared activity during trials in which specific behaviours occurred against those in which they did not. Example neurons in both OFC and LPFC (figure 7*a,b*) showed strong modulation by video content. To quantitatively assess neuronal sensitivity to video content while accounting for our large number of correlated regressors, we employed the firing rate model described in equation 2.4 fitted to the firing rate data via GLM with elastic net regularization (figure 7*c* and figure 1). This model recovered the large firing rate modulations for our example units while also discovering a number of additional ethogram and gaze behaviour regressors that modulated the activity of these example cells. In particular, the LPFC example neuron was modulated by the presence of multiple monkeys within the scene (count ≥1), camera movements (camera zooming out, camera tracking, camera panning, and camera slewing), foraging behaviours (e.g. drink, ingest food, and chew), and agonistic behaviours (joint aggression, submit). Firing rates declined when the subject monkey looked away from the screen (gaze offscreen), which is consistent with the overall lower firing rate associated with less visual stimulation in the blank screen condition for this neuron. Nevertheless, the activity of this neuron was also negatively modulated by the parameter ‘proportion gaze offscreen’, suggesting that some features of the presented videos that made monkeys less likely to choose to direct their gaze at the screen (features which were not otherwise well described by the ethogram) also produced an overall lower firing rate for this cell.

As with the PSTHs, the model result revealed considerable heterogeneity in the population of PFC units. Because assigning p-values to individual regressors is inappropriate for elastic net regularization, in order to estimate the false discovery rate associated with our model we performed a permutation test, according to which, for each unit, the relationship between the spike count in each bin and the ethogram regressors was randomly permuted, and the model then run on the resulting data. This permutation test identified no non-zero regressors for any unit, implying a false discovery rate of exactly zero. While this cannot rule out that some regressors were falsely identified as modulating firing rate due to their correlation with other regressors which were the true cause of the effect, the result of this permutation test speaks to the robustness of the elastic net regression technique.

To better understand the representation of ethogram and gaze variables in the population of PFC units, we counted the number of units exhibiting non-zero coefficients for each regressor as well as the number of regressors with non-zero coefficients for each unit (figure 1). These results reveal a few notable trends. Overall, the gaze-related regressors appear to be comparatively well represented in both OFC and LPFC. However, a higher proportion of OFC units responded to a higher proportion of viewed behavioural categories in comparison to LPFC (figure 1). The overall proportion of units sensitive to each viewed behaviour was fairly uniform across the set of behaviours that we investigated, with the exception of ‘grasp food', ‘manipulate food', ‘ingest food' and ‘ingest grooming manipuland', each of which appears to be less well represented than most of the other behaviours. There are a few possible explanations for this. One possibility is raised by noting that these are all comparatively specific behaviours and nested within ‘foraging', and their lesser degree of representation in the population may reflect the relative specificity of their definitions. Another common feature among these behaviours is that they all tend to be relatively brief when they occur. With this in mind, the relatively low representation of these behaviours in the model results could reflect limitations in the assumptions that went into formulating the model. In particular, the model assumes that the units' firing rates will respond to each behaviour with a boxcar-like profile, lasting exactly as long as the behaviour is viewed, with a 100 ms lag. If the true temporal response profile differs from this assumption, the model is likely to under-detect true responses, and this effect would be more pronounced for shorter duration behaviours. However, the proportions of responses to ‘attack' and ‘submit', behaviours which also tended to be relatively brief, were more in line with the rest of the ethogram.

## 4. Discussion

Our study presented monkeys with an information foraging task, with a simulated ‘environment' consisting of naturalistic, information-rich videos of a diverse set of conspecific behaviours. During the task, monkeys made short-scale orienting decisions, ‘micro-decisions’ about where to orient their gaze during the video presentation, and longer scale presentation decisions, ‘macro-decisions' about what kinds of videos to view. Monkeys' presentation decisions revealed a strong preference for viewing videos of conspecifics over viewing a blank screen and a strong preference for less predictable videos over more-predictable videos. Contrary to our expectations, the nature of the viewed videos did not impact monkeys' presentation decisions. Several possible explanations may account for this result. First, monkeys simply may have had no preferences among the various behavioural categories in the ethogram. The striking effect of viewed behavioural categories on monkeys' orienting decisions would seem to belie that explanation, but it is possible that these are fundamentally different decision processes, with distinct, unrelated preference functions. A related possibility is that monkeys did in fact make presentation decisions based on the viewed information, but our ethogram failed to capture the relevant dimensions of the stimuli. Again, this is at odds with the ethogram's success in explaining at least some of the variability in gaze behaviour and neural activity. However, monkeys' very strong preference for the Switch option over Continue or Repeat suggests that the lack of preferences for specific behaviours may have been a consequence of the nature of the ‘foraging environment' that we presented them with. Although our relatively large video database meant monkeys saw the same video sequence within the same behavioural session relatively infrequently, all of the subject monkeys performed this task regularly over many months and consequently were eventually exposed to the entire video database numerous times. If monkeys were able to quickly recognize a given video sequence and generally preferred the relative novelty (or at least unpredictability) offered by Switch, there may have been little utility in continuing or repeating a given video which the monkey already remembered well. Furthermore, even if monkeys had weak preferences for certain kinds of information, it is possible that the cognitive load of forming a decision on the basis of the viewed behaviour incurred a cost greater than the value offered by the information, and so monkeys' optimal strategy was to rely on simpler decision heuristics.

Our measures of aggregated gaze behaviour, gaze onscreen and gaze typicality, identified times within each video stimulus in which strong attractors of gaze occurred. Comparing these measures to our ethogram allowed us to identify viewed features and behaviours that were highly salient to the monkeys. The visual motion caused by camera movements, but also social information from aggression, attract the monkeys' gaze towards the screen (figure 6*a*), while biological and social elements such as faces, genitalia and submissive displays reliably attract gaze (gaze consistency, figure 6*b*) from the viewing monkeys. This suggests a ‘bottom-up' mechanism which causes a rapid, potentially involuntary shift of attention to the screen, and a subsequent top-down mechanism that turns the attentional spotlight onto elements of high social or biological value.

Neurons in the PFC have been known to be involved in visual categorization, hence it is no surprise to find that neurons in LPFC and OFC are found to have a robust response upon video presentation [37,38]. Interestingly, many OFC neurons also displayed a large phasic response at the end of video playback (figure 4). While our experimental design is not capable of definitively explaining this, we suspect that the end of video playback served as a reliable and temporally proximate cue predicting the upcoming juice reward at the end of the trial. Because OFC neurons signal reward expectancy [39], this may be the source of the population-level activity we observed. While more neurons in OFC appear to be modulated by a higher fraction of ethogram regressors, we do not find significant differences in the types of factors associated with changes in activity in OFC and LPFC. Furthermore, we found considerable heterogeneity in both populations (figure 1), showing modulatory effects on neuronal firing in both directions to the same feature element in the videos, suggesting a sparse representation of viewed behavioural categories in OFC and LPFC.

Damage to the OFC in human patients results in deficits in social valuation, emotional behaviour and decision making [40–42]. Studies carried out in non-human primates have shown responsiveness of individual OFC neurons to faces [43,44] and distinguished images in biologically relevant social categories twice as readily as representing juice volume [13]. Furthermore, Sliwa *et al*. [22] used fMRI to demonstrate that OFC is a part of a network which is selectively engaged during observation of social interactions compared to interacting objects. This is consistent with our finding that the categories of viewed behaviours are more strongly represented by units in OFC than in LPFC (figure 1). Our results also demonstrate that this increase in activity is not simply a global effect of heightened engagement, but that individual types of behaviour are processed differently by individual neurons. This is highly suggestive that these detailed representations are playing an important computational role in the function of OFC during observation of social interactions. Previous studies have also shown evidence that single neurons in OFC encode common-currency value representations when deciding between stimuli predicting juice rewards of different flavours and volumes [5,6] while neurons in LPFC tracked the subsequent choice, actioned by a saccade, to juice consumption [8]. Here, we do not find a particularly strong action-based representation in the LPFC during the 2AFC phase, suggesting that this representation is at least partially task dependent. However, during the FV phase, monkeys made numerous short-latency decisions to reorient their gaze focus, with a commensurately increased frequency of individual LPFC units' activity correlating with the gaze metrics (figure 1).

In recent years, there has been a growing appreciation for the limitations of what can be learned about the brain by studying its relationship to behaviour only within the context of highly artificial tasks in highly constrained contexts. The use of naturalistic video stimuli to expose non-human primates to a visual context with increased external validity has been invaluable for improving our understanding of both natural vision (e.g. [45]) and social cognition (e.g. [46]). However, the increased complexity of the stimulus compared to still images or abstract figures introduces its own limitations and demands an approach to data analysis and interpretation that relies heavily on statistical controls rather than experimental controls. In our case, the relatively free nature of both the task and the stimulus set (e.g. animals were not required to fixate during video playback; stimuli were not explicitly counterbalanced for low-level visual features) make this approach relatively poorly suited for certain aspects of higher order visual information processing. Decision making in the context of naturalistic scene viewing adds a further level of complexity. Here we have endeavoured to take an approach emphasizing naturalism and external validity in our experimental design. Our analysis is based on an ethological understanding of primate behaviour, to group data across multiple viewings of different manifestations of the same underlying behaviour. A major challenge with this approach is in producing a comprehensive and accurate record of the behaviours within the video stimuli that can be used as regressors for statistical analysis of neuronal activity. However, with the rapid advance of computer vision and machine learning approaches to video categorization, we expect it will soon become feasible to apply this strategy with larger and larger stimulus sets using automated or semi-automated video scoring. Ultimately, we envision a near future in which such an approach will make it feasible to comprehensively describe both the behaviour and environmental context of animals engaged in entirely unconstrained natural behaviour, alongside neurophysiological data, providing a powerful tool for understanding the natural brain.

## Supplementary Material

Figure S1: Autocorrelation coefficient for example neurons used for selection of window size for spike rate estimation

## Supplementary Material

Example of stimulus video with collected gaze information

## Data accessibility

Data and code for analysis are available at the following URL: https://adams-2020-data.s3.amazonaws.com/surfer-full-dataset.zip.
