# Supplementary material for: Neurons in primate prefrontal cortex signal valuable social information during natural viewing
Source: Philos Trans R Soc Lond B Biol Sci. 2021 Jan 11;376(1819):20190666. doi: 10.1098/rstb.2019.0666 (PMC7815429; doi:10.1098/rstb.2019.0666)

Supplementary Information

# Figure S1: Autocorrelation coefficient for example neurons used for selection of window size for spike rate estimation


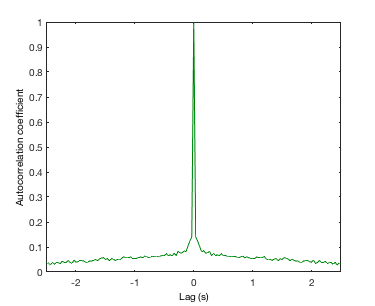

Supplement: Figure S1: Autocorrelation coefficient for example neurons used for selection of window size for spike rate estimation [file rstb20190666supp1.docx]
